# The Travellers' Exhibition

**Published:** 1903-05-16

**Authors:** 


### THE TRAVELLERS' EXHIBITION.

THE exhibition in connection with the Livingstone College which was held in the Examination Hall of the College of Physicians and Surgeons on the 6th inst., did not occupy a large space, although the exhibits were fairly numerous, because the chief intention of these exhibits was to show how much in the way of food, medicine, cooking utensils, and travelling equipment could be put in a small space and packed in a portable form. Models of tents and sections of "insect-proof" roofing materials were sufficient to indicate the styles of these articles, but all the other exhibits were the full size of the different commodities. Among foods were Messrs. Brand's specialities, including a new fever food, composed of meat essences combined with cream and eggs, which tastes rather like custard, and supplies the mixed proteid diet required for wasting diseases. Very palatable also is their preparation of beef-broth, which would come as a welcome change from beef-tea, and can be eaten as a jelly or made into a pleasant drink with hot water. The savoury meat lozenges form, perhaps, the most compact form of food possible, but almost equally convenient are the little packets of concentrated beef-tea.

Another beef preparation was shown by Messrs. Parke, Davis and Co. This is Mosquera's beef jelly, in which the nutrient properties of the beef are drawn out and predigested by a ferment obtained from the juice of the pineapple. It is claimed that Mosquera's preparation contains 90 per cent. of nutriment, of which 77 per cent. is albuminoid matter and 13 per cent. fat. The preparation is put up as a "beef meal" and "beef cacao," as well as a jelly and a liquid extract. The exhibit of Plasmon products was very popular, and a great many people were able to convince themselves practically that the addition of Plasmon to any article of food does not in the least alter the flavour or give any medicinal taste.

Among disinfectants Izal and Chinosol had representative displays of their various preparations. The Chinosol Company make a speciality of putting up the disinfectant in tablets of different strengths, which are very convenient in travelling, while the Izal soaps, ointment, and tooth powder are very useful preparations.

Among the etcetera of travel may be mentioned a very light, strong, and handy cooking-stove, made by Messrs. William Poore and Co., 155 Cheapside, which will burn any fuel, and though it contains a good-sized oven and three lids in the hot-plate, besides an open fire where chops or bacon could be toasted, weighs only 36 lbs. The same firm shows a useful tea-bottle, neat and portable, though it has a spirit-lamp in its lower part, which should be popular with working men and tourists as well as with the missionaries for whose benefit it was exhibited. The Jaeger, Wolsey, and Petanelle Companies showed samples of clothing, both underwear and outer garments. The Petanelle garments were so soft and fine that it was difficult to realise that peat fibre formed a part of them. One of their latest products is a peat and silk waterproofed motor hood, which should become popular. Only one typewriter was shown—the Blickensderfer—which surpasses most others in lightness, and is therefore well suited to the use of travellers.

In the afternoon Dr. Sambon gave a lecture on the diseases of tropical countries—malaria, yellow fever, etc.—dwelling on their parasitic origin, and giving an account of the life history of a disease organism. By way of preventive, however, he had nothing more novel to suggest than the hope that we might at some future time be able to discover and cultivate other parasites which should prey upon and devour the organisms of the disease, and in the meantime should avoid the poison-laden bites of mosquitoes, and the dangers of food which flies, which might be defiled by their previous resting-place, had touched. But within the necessary limits of a popular lecture Dr. Sambon was interesting and suggestive, and he impressed on his hearers the necessity for sanitary precautions by pointing out how every improvement in locomotion, every new development of trade, made easier the transmission of disease germs from their native habitat to some new soil.

The exhibition should prove useful in bringing before the public the claims of the Livingstone College at Leyton, which, founded in honour of the great missionary and explorer, gives to intending missionaries such a training in matters of health as will enable them to safeguard their own health and that of their families in every climate, and will tend to make them useful advisers to those among whom it is their lot to work. In the Year Book of the College are given selections from letters from old students, who detail their experience of various kinds of clothing, food, etc., which they have tried. It is to be regretted that last year's accounts show a deficit of £651, for which subscriptions are asked, as well as for an increased maintenance fund, and contributions towards a sum of £3,248 to purchase the College premises. The institution certainly has a claim on all who feel that the extension of the Empire implies an extension of our national responsibilities towards the natives of the new lands which we acquire. And its objects must appeal to all who think that a missionary should carry the gospel of sanitation into the dark places of the earth along with that other gospel which he goes forth to teach.

